# Prevalence of malaria, typhoid, toxoplasmosis and rubella among febrile children in Cameroon

**DOI:** 10.1186/s12879-016-1996-y

**Published:** 2016-11-08

**Authors:** Olivia A. Achonduh-Atijegbe, Kenji O. Mfuh, Aristid H. E. Mbange, Jean P. Chedjou, Diane W. Taylor, Vivek R. Nerurkar, Wilfred F. Mbacham, Rose Leke

**Affiliations:** 1 The Biotechnology Centre, University of Yaoundé I, Yaoundé, Cameroon; 2 North Pacific Global Health Program, University of Hawaii, Hawaii, USA; 3 Department of Tropical Medicine, Medical Microbiology and Pharmacology, John A. Burns School of Medicine, University of Hawaii, Hawaii, USA

**Keywords:** Fevers, Children, Malaria, Toxoplasmosis, Typhoid, Rubella, RDTs

## Abstract

**Background:**

The current roll-out of rapid diagnostic tests (RDTs) in many endemic countries has resulted in the reporting of fewer cases of malaria-attributed illnesses. However, lack of knowledge of the prevalence of other febrile illnesses and affordable diagnostic tests means that febrile patients are not managed optimally. This study assessed the prevalence of commonly treatable or preventable febrile illnesses in children between 6 months and 15 years using rapid diagnostic tests at the point-of-care.

**Methods:**

Febrile children were enrolled between February-April 2014 at a health facility after obtaining informed consent from parent. Eligible participants were aged 6 months-15 years with a history of fever in the last 24 h or axillary temperature ≥38 °C at consultation. All participants were tested using RDTs for malaria, typhoid, toxoplasmosis and rubella. Malaria parasites were further identified by microscopy and PCR. Clinical and household characteristics were recorded and association with pathogens determined.

**Results:**

Of the 315 children enrolled, the mean age was 5.8 ± 3.8 years. Stomach pain (41.2 %) was the most reported symptom. Prior to attending the health facility, 70.8 % had taken antipyretics, 27.9 % antimalarials, 11.4 % antibiotics and 13.3 % antifungal drugs. Among 315 children with fever, based on RDTs, 56.8 % were infected with malaria, 4.4 % with typhoid, 3.2 % with acute toxoplasmosis, and 1.3 % with rubella (all positive for rubella were in the same family and not vaccinated). All non-malarial infections were co-infections and approximately 30 % of the fever cases went un-diagnosed. Malaria prevalence by microscopy and PCR was 43.4 and 70.2 % respectively. The sensitivity and specificity of RDTs for the diagnosis of malaria were 75.98 and 100 % respectively, with 0.73 measurement agreement between RDTs and microscopy while that of RDT and PCR were 81 and 100 % respectively with a K value of 0.72. The use of Insecticide Treated Bednets was 44 %. There was a significant association between ITN non-usage and malaria (*p* = 0. 029) as well as drinking water and presence of typhoid (*p* = 0.047). No association was observed between type of housing and malaria, or toxoplasmosis and raising cats.

**Conclusion:**

Though malaria still remains the major cause of fever in children, using RDTs for other treatable febrile illnesses like typhoid and toxoplasmosis could facilitate the optimal management of febrile illnesses in children especially when these occur as co-infections with malaria

**Electronic supplementary material:**

The online version of this article (doi:10.1186/s12879-016-1996-y) contains supplementary material, which is available to authorized users.

## Background

Fever is the most common symptom of infectious diseases in tropical countries. A wide range of treatable or preventable pathogens are known to cause fever in patients who present with malaria-like symptoms, but do not have malaria [1]. Malaria continues to be the major cause of mortality in Cameroon and accounts for 35–40 % of all deaths, with 50 % of morbidity among children under the age of five with a considerable drop in morbidity observed between 2008–2011 [2, 3]. Malaria diagnosis in Cameroon has been reported to be predominantly presumptive leading to over diagnosis and over prescription of antimalarials [4]. Consequently, at least 50 % of most febrile patients are considered to have malaria and are given treatment out of fear of missing life-threatening *Plasmodium falciparum* infections. Serious consideration of other etiologies may not occur unless there is no clinical response to antimalarials treatment.

Many malaria endemic countries including Cameroon have adopted the WHO evidence -based guidelines for effective case management of malaria and rapid diagnostic tests (RDTs) have been presented as a means to realize this key strategy [5]. The recent roll-out /scale up of RDTs for malaria has highlighted the decreasing proportion of malaria-attributable illness in endemic areas [5–8] and the proportion of patients with non malarial febrile illnesses is likely to continue increasing with further adaptation of malaria RDTs [5–8]. However, the lack of affordable diagnostic tests and knowledge of the prevalence of other infectious diseases means that febrile patients are not managed optimally. These factors may promote the development of antimalarial and antibiotic resistance and unnecessary morbidity and mortality.

Diagnosis of non-malaria febrile illness in resource limited settings remains challenging [9]. With malaria RDTs, current treatment algorithms leave healthcare providers at odds on how to treat non-malaria febrile cases when RDTs are negative. The importance of differential diagnosis of febrile illnesses has been reported in many malaria endemic countries [10–21]. Typhoid fever is widely recognized as a major public health problem in most developing tropical countries and share similar symptoms with malaria [16], while toxoplasmosis is a cosmopolitan infection, which appears to be overshadowed in the tropics by other endemic diseases such as malaria and HIV [22].

With the successful contribution of RDTs as a key strategy in evidence-based malaria case management, RDTs, designed for peripheral health facilities have been developed or are being developed for other tropical infections including typhoid, toxoplasmosis, to improve patient and epidemiological surveillance. Most RDTs for non-malarial tropical infections are reported to rely on the detection of host antibodies against a single infectious agent and the sensitivity and specificity of host-antibody detection tests are both inherently limited [23]. However, point-of-care RDTs for typhoid, toxoplasmosis and rubella have been shown as useful tools for rapid diagnosis of these preventable infections in tropical countries [24–27].

A number of infectious diseases such as typhoid fever, rickettsial and arboviral infections have been recognized among febrile adult patients attending health facilities in Cameroon [19–21] while toxoplasmosis and rubella infections have mostly been reported amongst pregnant women [28–30]. However, limited information is available on the prevalence of febrile illnesses in children. Moreover, the proportion of children with fevers has not changed despite the reported drop in malaria morbidity in Cameroon [3]. This study assessed the prevalence of commonly treatable or preventable febrile illnesses in children between 6 months and 15 years using rapid diagnostic tests at the point-of-care.

## Methods

### Study design

The study was carried out in Nkolbison Health District, Yaoundé from February-April 2014. Nkolbisson is located in Mfoundi Division of the Centre Region of Cameroon, about 6 km from Yaoundé City Centre. Yaoundé is the capital of Cameroon with an estimated population of 2.5 million people. Malaria transmission in Yaoundé is perennial with two main seasons: the long wet season from February to November (with more intense rains between September and November) and a short dry season from June to July and December to January [31].

The target population was children between 6 months-15 years who came for outpatient consultation at the health facility. Children of both sexes were eligible if they were within the age limit, had a history of fever in the preceding 24 h or axillary temperature ≥38 °C on consultation, and the parent /guardian gave a written informed consent for his/her child to participate in the study. Since information on the prevalence of toxoplasmosis, rubella and typhoid for children with fever was not known, sample size was calculated based on malaria morbidity in children (38 %) as indicated in the National Malaria Control Program (NMCP) report in 2011 [3] taking into consideration the resources for the study .

### Patient and household characteristics

For all participants who met the inclusion criteria at the outpatient department of the health facility, a baseline questionnaire was administered to collect information on participants and the characteristics of their households. Data included: demographic data, signs and symptoms, type of medication taken prior to health facility visit, type of housing, main source of drinking water, ownership and usage of insecticide treated bed nets (ITN), and type of domestic animal in household.

### Sample collection and processing

A total of 2 ml of whole blood was collected from each participant. Of these, half of the blood was used to perform the malaria RDT on site, prepare thick blood films for microscopy, determine hemoglobin level by URIT 3200 automated hematology analyzer (URIT Medical, China) and ~ 20 μl spotted on filter paper (Whatman No.3) for DNA extraction and molecular studies. The rest of the blood was centrifuged and the plasma used to perform RDTs for typhoid, toxoplasmosis and rubella. Plasma was also aliquoted into cryogen vials, transported on ice packs to the Research Laboratory at the Biotechnology Centre and stored at -20 °C.

### Rapid diagnostic testing for malaria

The Malaria Ag Pf/Pan RDT (Standard Diagnostics Inc, South Korea) was used for malaria diagnosis. The Malaria Ag *Pf*/Pan antigen rapid test is a qualitative and rapid immuno-chromatographic test for the differential diagnosis of *P. falciparum* histidine rich protein II (*P.f* HRP-II) and lactate dehydrogenase (pLDH) common to *P. falciparum, P.malaria, P.ovale and P.vivax* (Pan). Briefly, 5 μl of whole blood was placed in the sample well, 4 drops of diluent added and results read in 15 min. The test was considered positive if there was a red line on the control (C) and test line (*P. falciparum*) or C and two test lines (*P. falciparum* or mixed *P. falciparum* and other species) or C and test line (non-*P. falciparum*) and negative when only the C line was observed.

### Rapid diagnostic testing for typhoid

Diagnosis of typhoid was done using the *OnSite* Typhoid IgG/IgM Combo rapid test (CTK Biotech Inc, USA). The *OnSite* Typhoid IgG/IgM Combo rapid test cassette is a lateral flow chromatographic immunoassay that can detect and differentiatiate of IgG and IgM antibodies to -*Salmonella typhi* and S. *paratyphi* in human blood. Briefly, one drop of plasma (50 μl) was added to the sample well, immediately followed by one drop of sample diluent. Results were read in 15 min. The appearance of a burgundy colored band in the C (control) line and in the M or G lines was considered to be positive for IgM and IgG antibodies, respectively. Meanwhile the absence of a coloured band in both the G and M lines, but present in the C line was considered negative.

### Rapid diagnostic testing of toxoplasmosis

Diagnosis of toxoplasmosis was done using the *OnSite* Toxo IgG/IgM rapid test (CTK Biotech Inc, USA). The OnSiteToxoIgG/IgM rapid test simultaneously detects and differentiates of IgG and IgM anti-*Toxoplasma gondii* antibodies in human serum or plasma. A total of 50 μl of plasma was placed into the sample well and a drop of sample diluent added and the results read within 15 min. The test was negative if only the control band (C band) developed and no burgundy colour observed in the both Test bands (T1 and T2) indicating the absence of anti- *T. gondii* antibodies in the specimen. Meanwhile, a positive test was indicated by the presence of a C band and a T1 band (IgM anti-*T. gondii*) or the presence of C and a T2 band (IgG anti-*T.gondii*) or the presence of both C band, T1 and T2 bands.

### Rapid diagnostic testing of rubella

Diagnosis of rubella was carried out using the SD BIOLINE Rubella IgG/IgM rapid test (Standard Diagnostics Inc, South Korea). The SD BIOLINE Rubella IgGIgM kit is intended to indicate the immune status or confirm recent rubella infection. Five microlitres (5 μl) of sample were placed into the sample well, 4 drops of diluent added and results read within 30 min. A test was positive if two pink lines C (Control) and G (IgG) or C and M (IgM) or three pink lines C, G and M developed in the result window. A negative test was indicated by one pink line C in the result window.

### Malaria microscopy

Thick films stained with 10 % Giemsa were used for definitive parasite counts; 200 high power fields were screened before a slide was declared negative. The number of parasites per 200 leucocytes was recorded and converted into parasite density per μl by assuming an average white blood cell count of 8000/μl. The mean of 2 slide readings from 2 independent readers was performed and discrepancies greater than 10 % were performed by a third reader (microscopy quality assurance expert).

### *Plasmodium* speciation by PCR

DNA was extracted from dried blood spot on filter papers from each participant by chelex boiling method as described by [32]. The 18srRNA gene was amplified as described by [33] using a T3 thermocycler (Biometra, UK). The amplification was performed following a two round PCR consisting of the genus specific amplification (conserved sequences) with a first set of genus primers (outer PCR) followed by the species-specific amplification (inner PCR). The products were electrophoresed on 2 % agarose gel stained with ethidium bromide and visualized over an ultraviolet transilluminator.

### Data analysis

Data were analysed using SPSS version 16.0 statistical software. Descriptive statistics were used to determine the prevalence of each of the pathogens and past infections. The sensitivity, specificity, positive and negative predictive values of RDT versus microscopy and PCR for malaria diagnosis were determined using Epi Info version 7 and the 2- Tailed P exact Fisher test used for comparisons. Crude odds ratio (ORs) with their 95 % confidence intervals (CI) were estimated for association between household characteristics (type of housing, major source of drinking water, ITN usage, cat ownership and the prevalence of pathogens. A modern house was defined as a house with corrugated roof, cement plastered floors and walls, electricity and pipe borne water. P-values of < 0.05 were considered statistically significant. Diagnostic performance of each of the RDTs used as reported by the manufacturer is indicated in Additional file 1.

## Results

### Clinical and household characteristics of study participants

Of the 315 children enrolled in the study, the average temperature at enrolment was 37.9 ± 1.3 °C. Among the children, 49.8 % were male and the average age was 5.8 ± 3.8 years. Mean haemoglobin levels were 11.0 ± 7.2 g/dl, with 9.6 % of the children being anemic (Table 1). Of the symptoms commonly found in febrile children, stomach pain (41.2 %), headache (21.9 %), cough (18.1 %) and diarrhoea (10.5 %) were the most common, while respiratory difficulties (1 %) was the least prevalent (Table 1). Eighty seven percent (87 %) of the study participants had taken at least one type of medication prior to visiting the health facility, including, antipyretics (70.8 %), antimalarials (27.9 %), antibiotics (11.4 %) and antifungals (13.3 %).

**Table 1:** Clinical characteristics of the study participants at enrollment

Variable	Value^a^
Number of males	157 (49.8 %)
Number of females	158 (50.2 %)
Mean age (years ± SD)	5.8 (±3.8)
Mean weight (kg ± SD)	21.4 (±11. 4)
Mean temperature (°C ± SD)	37.9 (±1.3)
Mean hemoglobin (g/dl ± SD)	11.0 (±7.2)
Mean weight (kg ± SD)	21.4 (±11. 4)
Diarrhoea/dysentery	33 (10.5 %)
Joint pains	6 (1.9 %)
Headache	69 (21.9 %)
Cough	57 (18.1 %)
Common cold	27 (8.6 %)
Vomiting	57 (8.1 %)
Stomach pain	132 (41.9 %)
Rashes	5 (1.6 %)

^a^ *Data for all 315 children was included in the calculations*

For household characteristics, 51.5 % of the participants lived in a modern house with 36.5 % living in an unfinished modern house (Table 2). The major source of drinking water was a borehole/ well (41.9 %) (Table 2). Although 79.7 % of the study participants own an insecticide treated bednet (ITN), only 44.8 % slept under an ITN the night prior to health facility visit. Twenty seven percent (27 %) of participants reported that they had a dog, cat or both (Table 2).

**Table 2:** Household characteristics of febrile children

Variable	Group	Number of Participants (%) *n* = 315
Type of housing	Completed Modern House (cement or ground)	161 (51.1)
	Unfinished Modern House (cement or ground)	115 (36.5)
	Poorly Constructed Houses with blocks	23(7.3)
	Poorly Constructed Houses Local material like planks	16 (5.1)
Main source of drinking water	Piped water into dwelling (e.g. water tap inside the house)	8 (2.5)
	Piped water into yard / plot (e.g. water tap in the yard)	14 (4.4)
	Borehole or Well	132 (41.9)
	Surface water (river or stream)	54 (17.1)
	Public tap	88 (27.9)
	Bottled water	19 (6.0)
Household owns an insecticide treated bednet (ITN)	Yes	251 (79.7)
	No	64 (20.3)
Patient slept under an ITN the night prior to health facility visit	Yes	141 (44.8)
	No	174 (55.2)
Household has cat(s)	Yes	39 (12.4)
	No	276 (87.6)
Household has dog (s)	Yes	47 (14.9)
	No	268 (85.1)

### Prevalence of malaria, typhoid, toxoplasmosis and rubella in febrile children

Prevalence of malaria, typhoid IgM, toxoplasmosis IgM and rubella IgM antibodies by RDTs was 56.8, 4.4, 3.2 and 1.3 % respectively (Table 3). Meanwhile, past infections of toxoplasmosis typhoid and rubella were 38, 4.4 and 4.1 % respectively. Co- infection of malaria with typhoid IgM, toxoplasmosis IgM or rubella IgM was 7.8, 5.6 and 2.3 % respectively, while approximately 30 % of the children were negative for all the four pathogens investigated. Only two of the children (0.006 %) tested positive for the four pathogens investigated. Malaria prevalence by microscopy and PCR was 43.4 % (136/315) and 70 % (221/315)) respectively. When microscopy was considered as gold standard,the sensitivity, specificity, positive and negative predictive values RDTs for the diagnosis of malaria were 75.98 % (95 % CI: 69.2–81.65), 100 % (95 % CI: 97.3–100), 76.0 % (95 % CI: 69.2–81.7) and 100 % (95 % CI: 97.3–100) respectively with 0.73 measurement agreement (K value) between RDT and microscopy. Meanwhile, when PCR was considered as gold standard, the sensitivity, specificity, positive and negative predictive values of RDT were 81 % (95 % CI: 75.3–85.6), 100 % (95 % CI: 96.1–100), 69.12 % (95 % CI: 60.9–76.3) and 100 %(95 % CI: 98.0–100) respectively with a K value of 0.72 (Table 4). Distribution of *Plasmodium* species in the study population were; *P. falciparum* 63.18 % (199/315), *P.ovale* 0.63 % (2/315), *P. malariae* 0.63 % (2/315), *P. falciparum* + *P.ovale* 5.4 % (17/315), *P. falciparum* + *P. malariae* 0. 32 % (1/315) and 29.84 % (94/315) were negative.

**Table 3:** Overall prevalence of pathogens in febrile children diagnosed by rapid diagnostic tests

Diseases/Pathogens	Number of children with recent infection (%)^a^	Number of children with past infection (%)^a^
Malaria	179 (56.8)	-
Typhoid (S*. typhi* and S. *paratyphi*)	14 (4.4)	14 (4.4)
Toxoplasmosis (*T.gondii*)	10 (3.2)	121 (38.3)
Rubella	4 (1.3)	12 (3.8)
RDT negative (all four pathogens)	108 (34.3)	-
Co- infections with malaria (*n* = 179)		
Malaria and typhoid (S*. typhi* and *paratyphi)* IgM	14 (7.8)	-
Malaria and toxoplasmosis (*T.gondii*) IgM	10 (5.6)	-
Malaria and rubella IgM	4 (2.3)	-
Malaria + typhoid (S*. typhi* and *paratyphi)* IgM + toxoplasmosis (*T.gondii*) IgM + rubella IgM	2 (0.006)	-

^a^ *Data for all 315 children was included in the calculations. Percentages of co-infections were calculated using the malaria infected children only (179). All non-malarial infections by RDTs were co-infections*

**Table 4:** Diagnostic Performance of RDT using Microscopy and PCR as gold standards for the detection of malaria parasites in febrile children

	Sensitivity % (95 % CI)	Specificity % (95 % CI)	NPV % (95 % CI)	PPV % (95 % CI)	Cohen’s Kappa	Degree of agreement (%)	*P*-value
Microscopy vs RDT	75.98 [69.2–81.65]	100 [97.3–100]	76.0 [69.2–81.7]	100 [97.3–100]	.73	86.35 [82.1–89.7]	0.000
RDT vs PCR	81 [75.3–85.6]	100 [96.1–100]	69.12 [60.9–76.3]	100 [98.0–100]	.72	86.7 [82.5–89.9]	0.000

PPV; Positive predictive value, NPV; Negative predictive value, RDT; Rapid diagnostic test, CI Confidence Interval, PCR; Polymerase chain reaction. The 2 -Tailed P exact Fisher Test was used to compare RDT with microscopy and PCR

### Association of household characteristics (risk factors) and pathogens investigated

An odds ratio analysis showed a significant association between ITN usage and *P. falciparum* infection where participants who slept under an ITN the previous night were more protected from malaria infection (OR = 0.77, 95 % CI = 0.460–1.290, *p* = 0.027) compared to those who did not use an ITN (Table 5). Meanwhile, there was no signification association between type of housing and malaria for participants who lived in uncompleted modern house (OR = 0.615, 95 % CI = 0.347–1.078, *p* = 0.072), poorly constructed house with blocks (OR = 0.636, 95 % CI = 0.147–1.624, *p* = 0.239), poorly constructed house with planks (OR =0.261, 95 % CI = 0.028–1.205, *p* = 0.064) compared to those who lived in modern houses. A significant association between main source of drinking water and typhoid was observed for participants who had a borehole or well (OR = 1.25, 95 % CI = 0.260–10.790, *p* = 0.047) compared to those with piped water into the dwelling while no significant association was found between acute toxoplasmosis and raising cats (*p* = 0.060).

**Table 5:** Odds ratio with 95 % confidence intervals for risk factors associated with *P. falciparum* infection in febrile children

Variable	Group	Crude Odds Ratio (95 % CI)	*P*-value
Type of housing	Completed modern house	Referent	
	Uncompleted modern house	0.615 (0.347–1.078)	0.072
	Poorly constructed house with blocks	0.636 (0.147–1.624)	0.239
	Poorly constructed house with local materials like planks	0.261 (0.028–1.205)	0.064
Slept under an ITN the previous night?	Yes	referent	
	No	0.770 (0.460–1.290)	0.029

Data from 221 febrile children that were positive for malaria by PCR was used. Other Plasmodium species (P.ovale and P.malariae) mostly occurred as co-infections with P. falciparum. ITN = Insecticide treated bednet, CI = Confidence interval

## Discussion

In this study, we determined the causes of commonly treatable or preventable febrile illnesses in children between 6 months and 15 years using rapid diagnostic tests at the point-of-care. All the 315 children in this study presented with fever and other symptoms that are common to malaria and other febrile illnesses like typhoid, toxoplasmosis and rubella thus confirming the need for a differential diagnosis of febrile illnesses as reported in many other malaria endemic countries [10–21]. Self- medication in this study population was high (87 %) and medication taken by patients prior to health facility visit were mostly antimalarial (27.3 %), antibiotics (11.4 %) and antifungal (13.3 %). This could act as a potential source of resistance to antimalarials or antibiotics in subsequent treatment. As such, there is need for continuous education of the population on the dangers of self medication, as self- medication can lead to unnecessary use of antimicrobials and life- threatening adverse events as reported in other studies [34, 35].

Increased malaria prevention and control measures are dramatically reducing the malaria burden in many endemic countries [36]. In Cameroon, malaria hospital morbidity was reported to have declined from 40.8 % in 2008 to 27.5 % in 2012 [3]. However, findings from the study described herein showed a high prevalence of 56 %, 43 and 70 % by RDTs, microscopy and PCR respectively. *P. falciparum* still remains the most prevalent malaria parasite species. This shows that malaria prevalence is still high amongst children in Cameroon and remains the main cause of fevers in this population despite the introduction of current control efforts such as the use of insecticide treated bednets (ITNs). Though RDTs were more sensitive compared to microscopy, and they were significantly less sensitive compared to PCR (Table 4). This confirms previous reports in similar settings that PCR is still the most sensitive test for malaria diagnosis [37]. Though RDTs are currently being used for routine diagnosis of malaria in health facilities, continuous monitoring and evaluation of these tests are necessary to ensure they remain effective for an informed policy decision on malaria control. Incorporation of a cost effective PCR in routine malaria diagnosis could be necessary in the long-term for effective malaria diagnosis. Insecticide treated bednet (ITN) usage has been reported to be protective against malaria in malaria endemic countries including Cameroon [38]. Although ITNs usage in this study was relatively low (44.8 %) compared to ownership (79.7 %), participants who did not sleep under the bed net were more susceptible to malaria infection (Table 5). There is need for the National Malaria Control Programme(NMCP) to intensify the sensitization of the population on the use of this important malaria control tool.

Typhoid and malaria share social circumstances which are important for their transmission, and so individuals in areas endemic for both diseases are at substantial risk of contracting both these diseases, either concurrently or an acute infection superimposed on a chronic one [16, 39, 40]. In this study, typhoid (*Salmonella typhi* and *S. paratyphi)* IgM antibodies seroprevalence, as diagnosed by RDTs, was 4.4 % and these patients presented with malaria—like symptoms and were also positive for malaria. These results were similar to the reports of Nsutebu et al., [19] who observed a typhoid prevalence of 2.5 % in a similar setting in Yaoundé but contradicts the work of Ammah et al., [20] who reported a prevalence of 26 %. These findings also support the report of Nsutebu et al.*,* [19] that typhoid fever is not as endemic in Cameroon as recently feared. All the typhoid cases recorded in this study occurred as co-infections with malaria. Risk factors such as the use of borehole or well as the main source of drinkable water was significantly associated with typhoid (*p* = 0.047). Public health measures such as improved personal hygiene and intensive community health education campaign [40] and the use of public water supply rather than wells could be useful for the reduction of typhoid prevalence in this population.

*Toxoplasmosis* infection in immunocompetent adults and children are usually asymptomatic or have symptoms that spontaneously resolve such as fever, malaise, and lymphadenopathy indicating a symptomless latent infection [41]. A report from the prevalence of toxoplasmosis in pregnant women in Cameroon showed a seroprevalence of toxoplasma IgG antibodies, IgG and IgM co-infection of 65 and 2.7 % respectively [28]. Febrile children in this study showed a seroprevalence of toxoplasmosis (*T.gondii*) IgM antibodies of 3.4 % and occurred as co-infection with malaria. Most of the toxoplasmosis infection observed in this study might have been congenital given the high prevalence previously reported in pregnant women [28] in a similar setting and the high prevalence (38.3 %) of past infections (IgG antibodies) observed in this study. These findings therefore, emphasize the need for a differential diagnosis of this preventable and treatable infection in febrile patients. Risk factors such as parents’ educational level and cats kept indoors have been reported to be associated with the acquisition of toxoplasmosis infection amongst primary school children [42]. However, no significant association was found between the raising of cats or dogs and toxoplasmosis IgM antibodies in this population. The lack of a significant association might have been due to a low prevalence of the disease, small sample size or the sensitivity/specificity of the RDTs used.

Rubella is a mild disease in children and adults, but can cause devastating problems if it infects the fetus, especially when the infection occurs during the first weeks of pregnancy [43, 44]. There is no specific treatment for rubella, but the disease is preventable by vaccination. A comprehensive vaccination program in most industrialized regions has reduced the incidence of the disease in these areas to low levels; vaccination is not carried out in many developing countries [45]. However, most children in Cameroon are vaccinated against rubella. The low seroprevalence of rubella IgM antibodies (1.3 %) observed in this study indicates the positive impact of the vaccination programmes. The 4 cases of rubella and malaria co-infection were from the same family whose parents reportedly did not believe in vaccines. Continuous community education may lead to complete elimination of this preventable febrile illness.

This study also has some limitations. First, the methods used in this study were unable to determine the cause of fever in approximately 30 % of the study population that were negative for all the four pathogens investigated. Investigations of other pathogens of viral and bacterial origin and biomarkers could provide additional useful diagnostic clues that may help to refine decision-making. Moreover, the RDTs for typhoid, toxoplasmosis and rubella maybe non-specific hence confirmation with more specific and sensitive tests such as cultures or PCR when available may be useful. Secondly, it is a health-facility-based study and it did not include patients within the community. Also, the influence of other factors such as the economic status of the family, education and the nutritional status of the children were not investigated.

Although the prevalence of typhoid, toxoplasmosis and rubella was low in this population, it will still be important to consider a differential diagnosis of these febrile illnesses so as to reduce overdiagnosis of malaria and overprescription of antimalarials.

## Conclusion

Though malaria still remains the major cause of fever in children, using RDTs of other treatable febrile illnesses like typhoid and toxoplasmosis could facilitate the optimal management of febrile illnesses at the point- of- care. Results from RDTs also provide useful information to health-workers on how to manage febrile patients at the point of care either when malaria is negative or exists as a co-infection with other infectious diseases such as typhoid and toxoplasmosis

## Additional file

Additional file 1: Diagnostic performance of RDTs used for diagnosis of commonly treatable or preventable febrile illnesses in children. Table shows the diagnostic performance of rapid diagnostic tests used for the diagnosis of commonly treatable or preventable febrile illnesses in children as reported by the manufacturer. (DOCX 13 kb)

## Abbreviations

CI: Confidence interval
ITN: Insecticide treated bednets
NMCP: National Malaria Control Programme
OR: Odds ratio
PCR: Polymerase chain reaction
RDT: Rapid diagnostic test
