# Supplementary material for: Prevalence of malaria, typhoid, toxoplasmosis and rubella among febrile children in Cameroon
Source: BMC Infect Dis. 2016 Nov 8;16:658. doi: 10.1186/s12879-016-1996-y (PMC5101675; doi:10.1186/s12879-016-1996-y)
Supplement: Additional file 1: — Diagnostic performance of RDTs used for diagnosis of commonly treatable or preventable febrile illnesses in children. Table shows the diagnostic performance of rapid diagnostic tests used for the diagnosis of commonly treatable or preventable febrile illnesses in children as reported by the manufacturer. (DOCX 13 kb) [file 12879_2016_1996_MOESM1_ESM.docx]

| **Rapid Diagnostic Test (RDT)** | **Sensitivity**  **(%)*** | **Specificity**  **(%)*** | **Overall Agreement (%)*** | **Reference method*** | **Manufacturer** |
| --- | --- | --- | --- | --- | --- |
| **Malaria Ag Pf/pan** | 99.7 (P.f), 95.5 (non-P.f) | 99.5 | - | - | Standard Diagnostic Inc |
| **OnSite Typhoid IgG/IgM Combo** |  |  |  |  |  |
| Typhoid IgM | 91.2 | 99 | 97.9 | Commercial EIA | CTK Biotech |
| Typhoid IgG | 92.9 | 99 | 99.0 | Commercial EIA | CTK Biotech |
| **OnSite Toxo IgG/IgM** |  |  |  |  |  |
| Toxoplasma IgM | 100 | 99.3 | 99.3 | Commercial EIA | CTK Biotech |
| Toxoplasma IgG | 91.6 | 99.0 | 98.5 | Commercial EIA | CTK Biotech |
| **SD BIOLINE Rubella IgG/IgM** |  |  |  |  |  |
| Rubella IgM | 98.33 | 97.64 | - | Rubella IgG/ IgM ELISA | Standard Diagnostics Inc |
| Rubella IgG | 99.14 | 91.55 | - | Rubella IgG/ IgM ELISA | Standard Diagnostics Inc |

**Supplement 1: Diagnostic performance of RDTs used for diagnosis of commonly treatable or preventable febrile illnesses in children**

**Data from manufacturers*
